# Puerperal Group A Streptococcal Infections: A Case Series and Discussion

**DOI:** 10.1155/2013/751329

**Published:** 2013-05-09

**Authors:** Mary T. Busowski, Melissa Lee, John D. Busowski, Kauser Akhter, Mark R. Wallace

**Affiliations:** Orlando Health, Orlando, FL 32806, USA

## Abstract

Puerperal group A streptococcal infections, a major postpartum killer during the late 19th and early 20th centuries, have become (fortunately) rare. We describe a cluster of 4 serious peripartum group A streptococcal infections occurring within the past five years at a single medical center. These cases were not epidemiologically linked and serve to illustrate the continuing risk of these potentially fulminant infections.

## 1. Introduction

Puerperal group A streptococcal (GAS) infections were a major cause of peripartum morbidity and mortality throughout the preantibiotic area. The incidence of these infections fell steadily from the 1940s to 1980s, coincident with the general drop in invasive GAS infections (necrotizing fasciitis, myositis, and streptococcal toxic shock) during that period. Unfortunately, serious puerperal GAS infections and other invasive GAS infections have reappeared over the past 25 years, and these infections must now be considered in the differential diagnosis of postpartum sepsis [1, 2]. Though still infrequent, such infections must be recognized and treated aggressively to prevent severe morbidity or mortality. We present our recent, single institution experience with 4 GAS peripartum cases occurring over a 5-year period, 3 of which were life-threatening puerperal sepsis and a fourth necrotizing cervicitis due to GAS. We review here relevant recent literature regarding this reemerging threat.

## 2. Cases

A previously healthy 24 y/o woman presented to a local emergency department two days after an uneventful vaginal delivery. On presentation, she was noted to be febrile with chills, tachycardia, and hypotension. Her condition quickly deteriorated, and she was transferred to our tertiary care center. She required aggressive vasopressor and ventilator support for severe sepsis with toxic shock syndrome. Gynecologic surgical management included a supracervical hysterectomy and bilateral salpingoophorectomy; the pathology revealed uterine bilateral fallopian tube necrosis and necrotizing endometritis. Multiple cultures including blood and endometrial tissue grew GAS. Her hospital course was complicated by multisystem organ failure, refractory shock requiring sustained heroic doses of vasopressor agents, and peripheral necrosis of the extremities. She survived with significant and permanent disabilities, including multiple amputations of the extremities.

A previously healthy 27-year-old woman developed fever, chills and hypotension two days after an uneventful vaginal delivery. She was transferred to the intensive care unit where she was treated for refractory septic shock with required vasopressor support and mechanical ventilation. Vaginal and blood cultures were positive for GAS, establishing an ascending (vaginal) route of infection. She was treated with ampicillin/sulbactam and clindamycin. She survived severe sepsis and was discharged after a 15-day hospital stay.

A 27-year-old woman presented seven days after an uneventful vaginal delivery complaining of burning abdominal pain; she was discharged after a normal vaginal ultrasound. She returned two days later complaining of left leg pain and was found to be in septic shock. An emergent exploratory laparotomy revealed two liters of purulent fluid and “angry, beefy red” fallopian tubes; the laparotomy was aborted due to profound acidosis and refractory hypotension. An intraoperative culture from the abdominal fluid was positive for group A streptococci. Her ICU course was complicated by multisystem organ failure. She returned to the operating room two days after her initial laparotomy for a total abdominal hysterectomy and bilateral oopherectomy. Histopathology of the uterus and fallopian tubes confirmed the intraoperative findings of extensive myometrial infarct and necrosis. The patient's subsequent ICU course was complicated by necrotizing fasciitis of her abdomen wall and thighs as well as multisystem organ failure with acute renal insufficiency, hepatic dysfunction, thrombocytopenia, and metabolic derangements. Following a surgical debridement of the soft tissues, the patient eventually stabilized and was discharged home after a lengthy inpatient stay.

A previously healthy 19-year-old, African American, primigravida presented at 27 weeks of gestation with a complaint of two days of progressive, severe abdominal pain and “leaking vaginal fluid.” The patient's past medical history was unremarkable. Her initial cervical exam revealed normal appearing external genitalia with a diffusely edematous cervix with ulcerated lesions and extensive cervical necrosis. Premature ruptured membranes were ruled out with negative nitrazine and fern testing. Cervical cultures revealed group A streptococci.

An exam under anesthesia was performed to better examine and describe the cervix. There were no external lesions, but there were extensive edema and necrosis of the posterior cervix. Histopathology confirmed acute necrotizing inflammation. The mucous surface of the endocervix was inflamed. On hospital day two, the patient passed a large amount of necrotic tissue. She continued to remain afebrile and had resolution of her abdominal pain. A repeat exam under anesthesia was performed on hospital day three and demonstrated a significant improvement in the appearance of the cervix. The remainder of her pregnancy was unremarkable, and she went on to deliver a term infant via vaginal delivery.

All four young women were HIV negative, nondiabetic, and free of chronic liver or renal disease. None had a significant premorbid medical history. In all cases, antibiotic therapy involved the use of a beta-lactam plus clindamycin.

## 3. Discussion

Severe peripartum GAS infections, a scourge of the pre-antibiotic area, virtually disappeared during the mid 20th century, but have been reported sporadically since the early 1990s. While most of the reports have been 1-2 unrelated cases [3–14], some larger collections of patients have been reported. Nathan et al. [3] studied 18 GAS isolates from puerperal infections in the Salt Lake City region between 1991 and 1997; no outbreak strain was identified. By contrast, Raymond et al. [15] described a cluster of 5 cases of puerperal sepsis in which 3 of the 5 infections were clonal. Our 4 cases were separated by 1 year, and there was no epidemiologic list between them or any known GAS infections in their health care workers.

Some epidemiologic work has attempted to quantify the incidence of GAS puerperal infections in the USA. Chuang et al. [16] used CDC data (1995–2000) on 87 postpartum GAS infections from selected US counties to estimate that ~220 cases per year of invasive GAS occur in the USA annually. The mortality rate of the 87 documented cases was 3.5%. Interestingly, they found that cases caused by identical emm types clustered more than chance alone would suggest, raising the possibility that some of these cases may have a common source. Aronoff and Mulla [17] extended the earlier work of Chuang et al. [16] in a study limited to Florida. They found that 4 cases of hospitalized invasive postpartum GAS between 1996 and 2000, which represented 1.6% of all reported invasive GAS infections reported during that period.

The dramatic predisposition of pregnant or postpartum women for significant GAS infections was delineated by Deutscher et al. [18], who found that postpartum women have a 20-fold increased incidence of GAS infections as compared to nonpregnant women. The reasons for this predilection are not clear. GAS vaginal carriage in late pregnancy is only 0.03%, so GAS is clearly not “normal” vaginal flora [19]. Selected Streptococcal M types, especially 28, are overrepresented in GAS puerperal sepsis, suggesting that bacterial properties, in addition to host factors and peripartum changes, contribute to the predisposition to serious GAS infection [20]. 

Severe nonobstetrical GAS infections are most often individual and community acquired but can be hospital acquired in as many as 12% of cases. In some instances health care workers can serve as the means of transmission between patients [21], and nosocomal outbreaks may occur. Familial transmission of a severe GAS clone has also been reported [22] and explains some community acquired cases. Though rare, health care workers may acquire GAS infection from patients or even cadavers [23].

The clinical features of our 3 cases of severe peripartum sepsis are similar to most prior reports in that the onset was within a few days postpartum, occurred in previously health women, and progressed rapidly from fever and abdominal/pelvic pain to vasopressor dependent shock recurring ICU support for multiorgan failure [3–5, 7–14, 24]. Patients may present with puerperal sepsis due to GAS after discharge from the hospital [19], as one of our patients did; clinicians from emergency department or outpatient settings must be open to the possibility of a life-threatening infection when postpartum women present with unstable vital signs, high fever, or an unexpected “toxic” appearance. None of the cases had any symptoms prior to the second postpartum day, suggesting they were not hematogenous in origin but rather arose from vaginal colonization [25, 26]. Our fourth case, cervical necrosis due to GAS without sepsis, illustrates that not all serious peripartum GAS infections are life-threatening, but all require careful evaluation and aggressive care.

The treatment of severe peripartum sepsis requires antibiotics coverage aimed at the likely causes: GAS, *S. aureus,* anaerobes, and *E. coli* [19]. Given that GAS has special potential to be a “bad actor,” the use of clindamycin to block toxin production is recommended in addition to a broad spectrum beta-lactam agent with good gram negative and anaerobic activity (a carbapenem, piperacillin-tazobactam, etc.). MRSA coverage may be needed initially pending cultures [19]. When GAS infection is probable or proven, the addition of intravenous immunoglobulin may be indicated. Though its utility in GAS associated shock is not as firmly established as clindamycin, it may be a key adjunct to care in critical situations [27].

The prevention of puerperal sepsis is problematic. The infection is rare, occurs generally in the health host, and may be fulminant. Screening for vaginal carriage of GAS is unlikely to be helpful as such carriage is rare [19]; however, there are at least 2 cases where GAS vaginal carriage was documented (and ignored) prior to the onset of GAS puerperal sepsis [12]. A vaccine, the optimal prevention, is not yet available. Strict infection control practices will serve to limit the occasional case due to spread between patients or from staff to patients [19].

The CDC has issued guidelines to prevent invasive GAS infections among household contacts of severe GAS cases and to limit hospital outbreaks [28]. Chemoprophylaxis is usually not offered to household contacts. Single cases of postpartum or postsurgical GAS, as described in the report, should lead to enhanced surveillance, while ≥2 postpartum/postsurgical cases demand a full epidemiologic investigation with cultures of the relevant health care workers.

For sporadic single cases, it seems that prevention must take a back seat to prompt recognition and aggressive management of this rare, but sometimes terrifying, infection, of the postpartum period. We hope our cases and this review of the relevant literature serve as a reminder of this emerging infection.
